# Urinary tract infection among people living with human immunodeficiency virus attending selected hospitals in Addis Ababa and Adama, central Ethiopia

**DOI:** 10.3389/fpubh.2024.1394842

**Published:** 2024-09-04

**Authors:** Ketema Bizuwork Gebremedhin, Engida Yisma, Haile Alemayehu, Girmay Medhin, Girma Belay, Shubhada Bopegamage, Wondwosson Amogne, Tadesse Eguale

**Affiliations:** ^1^College of Health Sciences, Addis Ababa University, Addis Ababa, Ethiopia; ^2^Aklilu Lemma Institute of Pathobiology, Addis Ababa University, Addis Ababa, Ethiopia; ^3^Allied Health & Human Performance, University of South Australia, Adelaide, SA, Australia; ^4^Faculty of Medicine, Slovak Medical University, Bratislava, Slovakia; ^5^Ohio State University Global One Health, Addis Ababa, Ethiopia

**Keywords:** human immunodeficiency virus (HIV), urinary tract infections (UTIs), antibacterial resistance, bacterial uropathogens, Ethiopia

## Abstract

**Background:**

Urinary tract infections (UTIs) and antibacterial resistance (ABR) are important public health problems, but they are not well-studied among people living with human immunodeficiency virus (PLHIV) globally, especially in low-income countries. Therefore, it is important to regularly measure the extent of UTIs and ABR in the most susceptible populations. This study aimed to investigate the prevalence of UTIs, associated factors, bacterial causal agents, and their antibiotic susceptibility profile among PLHIV in central Ethiopia.

**Methods:**

A hospital-based cross-sectional study was conducted to recruit 688 PLHIV by a simple random sampling method. Background information was gathered through interviews, while clinical information was gathered from recent information sheets of patient charts using organized, pretested, and validated study tools. Midstream urine was collected aseptically and transported to the Microbiology Laboratory of Aklilu Lemma Institute of Pathobiology within 4 h of collection, maintaining its cold chain. Standard conventional microbial culture methods and matrix-assisted laser desorption ionization-time of flight (MALDI-TOF) mass spectrometry were used to identify the bacterial isolates at the species level. Kirby Bauer’s disk diffusion method was used to determine the antibiotic susceptibility profile of the bacterial isolates based on the interpretation guidelines of the Clinical Laboratory Standard Institute. Logistic regression models were used to examine factors associated with the occurrence of UTIs among PLHIV attending selected hospitals in Addis Ababa, and Adama.

**Results:**

Out of 688 PLHIVs involved in the current study, 144 (20.9%) were positive for UTIs, whereas the majority were asymptomatic for UTIs. In the multivariable logistic regression analysis, only HIV RNA ≥ 200 copies/ml [AOR = 12.24 (95% CI, 3.24, 46.20), *p* < 0.01] and being symptomatic for UTIs during the study period [AOR = 11.57 (95% CI, 5.83, 22.97), *p* < 0.01] were associated with the occurrence of UTIs. The dominant bacterial species isolated were *Escherichia coli* (*E. coli*; *n* = 65; 43%), followed by *Enterococcus faecalis* (*E. faecalis*; *n* = 16; 10.6%) and *Klebsiella pneumoniae* (*K. pneumoniae*; *n* = 11; 7.3%). Over half of the *E. coli* isolates were resistant to antibiotics such as gentamicin (GM; *n* = 44; 67.7%), amikacin (AN; *n* = 46; 70.8%), nalidixic acid (NA; *n* = 42; 64.6%), ciprofloxacin (CIP; *n* = 40; 61.5%), and azithromycin (AZM; *n* = 45; 69.2%). All of the *K. pneumoniae* isolates (*n* = 11; 100%), (*n* = 6; 54.5%), and (*n* = 7; 63.6%) were resistant to [amoxicillin as well as amoxicillin + clavulanic acid], ceftriaxone, and sulfamethoxazole + trimethoprim, respectively. All the *Staphylococcus aureus* (*S. aureus*) isolates were resistant to cefoxitin, which implies methicillin-resistant *S. aureus* (MRSA).

**Conclusion:**

The high prevalence of UTIs and antibiotic resistance revealed in the current study needs public health interventions such as educating the population about preventive measures and the importance of early treatment of UTIs. Our findings also highlight the need to provide UTI screening services for PLHIV, and healthcare providers should adopt antibiotic stewardship programs to promote and ensure their appropriate and judicious use.

## Background

Urinary tract infections are highly prevalent worldwide, with approximately 150 million cases annually (1) among people of all ages; however, they are higher among youngsters, elders, and females (2). The infection poses significant public health concerns among PLHIV, mainly in developing countries (2, 3). This could be due to the suppressed immunity of the population and limited healthcare services in the region, which results in a high economic burden and reduced quality of life (3, 4). Untreated UTIs can lead to severe complications (5) such as pyelonephritis, renal failure, prostatitis, epididymitis, septicemia, endocarditis, osteomyelitis, septic arthritis, and meningitis (6, 7) which could be severe and sometimes life-threatening (6).

Worldwide*, E. coli* is the common bacterial causative agent of UTIs (2, 8–11). Other bacterial species such as *K. pneumoniae, Staphylococcus saprophyticus (S. saprophyticus), E. faecalis, group B Streptococcus, Proteus mirabilis (P. mirabilis), Pseudomonas aeruginosa,* and *S. aureus* also cause UTIs, albeit less frequently (2, 8, 10, 12). Rarely occurring bacterial species, such as *Bacillus cereus, Enterococcus hirae (E. hirae)*, *Staphylococcus* species (*sciuri, lentus, vitulinus, pulvereri*, and *xylosus*), *Salmonella species,* and *Acinetobacter,* are also implicated as causal agents of UTIs (2, 3, 10, 13–16).

The pathogenesis of the infection involves the bacterial coliforms inhabiting the urethral introitus and ascending into the bladder or kidneys (5), attaching to the urothelium with the help of their microbial adhesins, and binding to specific receptors on the host cells (2, 3, 5). Moreover, quorum sensing, a form of bacterial communication, plays a significant role in UTI pathogenesis. Bacteria communicate via signaling molecules to regulate gene expression, coordinate virulence factor production, and biofilm formation (2, 3). This helps sense population density and initiate actions, such as producing proteases, toxins, urease enzymes, and iron-scavenging systems, contributing to infection invasion and establishment of infection (3), which is manifested by flank pain, dysuria, pyuria, increased urinary frequency/urgency, and fever and chills if the infection involves the kidneys or when it is more severe (17). Furthermore, the diagnosis of UTIs is based on urine analysis for white blood cells and urine culture to identify bacteria and antibiotic susceptibility (18). However, antibiotic resistance challenges the effectiveness of antimicrobials, mainly in developing countries, due to inappropriate antibiotic use and poor antimicrobial stewardship programs (3).

UTIs represent a significant concern among PLHIV, with varying prevalence worldwide and in developing countries (19, 20). For instance, a systematic review and meta-analysis from Ethiopia revealed a 12.8% pooled prevalence of UTIs among PLHIV (20), which was supported by findings reported from the region, which ranges from 10.3 to 48.7% (4, 9, 21, 22). The burden of UTIs is impacted by the socio-demographic, background, and clinical characteristics of the population (4, 20, 23). The common causal agents of UTIs revealed by studies from developing countries, including Ethiopia, are *E. coli, S. aureus*, *K. pneumoniae, Enterococcus species,* and *Enterobacter aerogenes*, which is in agreement with global reports (9, 21, 24–26). Bacterial uropathogens among PLHIV usually demonstrated a higher rate of ABR (27), which is intense in low-income countries (27). Studies from low-income countries, including Ethiopia, revealed a higher rate of MDR, ranging from 45.9 to 95% (4, 19, 26–29). Thus, it is important to assess the extent of UTIs, and ABR associated with bacterial uropathogens, along with suggestions of possible interventions. Therefore, this study aimed to determine the prevalence of UTI, its associated factors, and the antibacterial susceptibility profile of the bacterial isolates among PLHIV attending selected antiretroviral therapy (ART) clinics in Addis Ababa and Adama, central Ethiopia.

## Materials and methods

### Study setting, and study design

A hospital-based cross-sectional study was conducted in central Ethiopia (Addis Ababa and Adama cities) among PLHIV from October 2022 to April 2023. Addis Ababa, the nation’s capital city, is located at a high altitude, 2,400 meters above sea level (30), while Adama is located 99 km southeast of Addis Ababa (31), in the great East African rift valley system, 08°32′29″N 39°16′08″E; 1712 meters above sea level (31). According to the world population review estimates, the population of Addis Ababa and Adama by the year 2024 would be 3.6 million and 213,995, respectively (32). According to the report from the Ethiopian Public Health Institute of 2023, among a total of 571, 119 adult PLHIV in Ethiopia, 107,496 were from Addis Ababa, while 6,300 were from Adama (33). In Ethiopia, 98% of healthcare settings, including all referral hospitals, provide comprehensive HIV care services (34). Thus, based on the level of comprehensive HIV care given in the hospitals, irrespective of UTI screening, four government Hospitals from Addis Ababa: Tikur Anbesa Specialized Hospital, Zewditu Memorial Hospital, Menilik the II Memorial Hospital, and Ras Desta Hospital, one public Hospital from Adama: Adama Comprehensive Hospital, and two private Hospitals from Adama: Sr. Aklesia Memorial hospital and Rift Valley University Hospital from Adama were included in the current study.

### Estimation of sample size and recruitment of study participants

The sample size was estimated using the formula [ $N=\frac{{\left(Z\right)}^{2}p\left(1-p\right)}{e^{2}}$ ], where *Z* is the standardized score at a 97% confidence interval (CI), e = margin of error, P = is the estimated proportion of the population with UTIs in the target community, and N = total estimated sample size. Thus, considering a 10.3% proportion (4), 97% CI, 0.03 margin of error, and 7.5% non-response rate, the total estimated sample size was 520 for the study population in Addis Ababa, and considering an 11.3% proportion (26), 95% CI, 5% margin of error, with the non-response rate (5%), and the total sample size calculated was 170 for Adama. The total sample size for the two cities was 688.

The studied populations were PLHIV attending ART clinics of selected referral hospitals providing comprehensive HIV care; The exclusion criteria include those who consumed two glasses of fluid an hour before attending the clinic, as fluid intake dilutes and reduces bacteria concentration in the urine (35), (b) received antibiotic treatment within a week before attending the clinic, which might affect UTI test result (36), (c) those with confirmed sexually transmitted diseases (STDs) who exhibit symptoms overlapping with UTIs (37), (d) pregnant or lactating mothers who might have immune suppression and an excess risk of having UTI (38, 39), menstruating women, could be due to the considerable lowering level of estrogen during the menstrual period (40), and the blood may contaminate urine samples which can affect UTI testing results, (e) individuals <18 years old, as this study focused on adult patients. Then, the estimated sample size was proportionally allocated across the selected seven ART clinics based on the total number of PLHIV attending the clinics during the study period, and a simple random sampling method was used to select study participants using the list of PLHIV within each clinic as a sampling frame.

### Data collection methods

Prospective background characteristics were collected through face-to-face interviews, while clinical characteristics were determined by reviewing recent information sheets of the patients’ charts. The meta-data and urine samples were collected using an expert-validated, pretested structured questionnaire adapted from related literature (26, 28), and checked for content validity index (CVI) rated at 0.82; indicating a high level of relevance, and appropriateness of the questionnaire in measuring the intended constructs. The data collectors were well-qualified nurses who received 2 days of training on research methodology, and how to collect the meta-data, and urine samples credibly. A total of 10 mL of midstream clean catch urine samples were collected in sterile urine cups with cover through precautions to prevent contamination and transported to the microbiology laboratory of Aklilu Lemma Institute of Pathobiology within 4 h of collection, maintaining its cold chain.

A loopful (0.01 mL) of well-mixed, serially diluted urine sample was inoculated onto plate count agar (HiMedia laboratory Pvt. Ltd., Mumbai, India) and incubated for 24 h at 37°C. When there was a delay in processing, the urine samples were stored at −20°C and cultured within 4 h. For study participants without symptoms of UTIs, a colony-forming unit (cfu) count of ≥10^5^/ml was considered positive for UTIs (41, 42), while for participants with symptoms of UTIs, a colony count of ≥10^2^cfu/ml (43) was considered positive for UTIs. Different microbiological media and biochemical tests (HiMedia laboratory Pvt. Ltd., Mumbai, India), such as triple sugar agar (TSA); lysine iron agar (LIA); Simmon’s citrate agar; urea, and tryptophan broth, eosin-methylene blue (EMB) and cystine–lactose–electrolyte-deficient agar (CLED) (41, 44, 45) were used to identify the Gram-negative bacterial isolates, while Gram-positive bacteria were identified using Enterococcus agar (EA) and mannitol salt agar (MSA) along with Remel Europe Ltd., Dartford, United Kingdom: potassium hydroxide (KOH); catalase and coagulase tests (41, 45). Finally, the presumptive bacterial isolates were then confirmed using the MALDI-TOF test (46).

The isolated Gram-negative bacteria were tested for 12 antibacterial agents, while the Gram-positive bacteria were tested for 18 antibacterial agents using the disk diffusion method and interpreted based on the clinical laboratory standard institute guidelines (64, 65). In total, 18 antibiotics with the disk potencies utilized (Sensi-Disks, Becton, Dickinson and Company, Loveton, United States): GM(10 microgram(μg)); streptomycin (S:10 μg); AN (30 μg); kanamycin (K:30 μg); NA (30 μg), amoxicillin + clavulanic acid (AMC:20/10 μg), amoxicillin (AMX:30 μg), ceftriaxone (CRO:30 μg), cephalothin (*CF*:30 μg), cefoxitin (FOX:30 μg), CIP (5 μg), sulfamethoxazole+ trimethoprim (SXT:23.75/1.25 μg), tetracycline (TE:30 μg), chloramphenicol (C:30 μg), AZM (15 μg), erythromycin (E:15 μg), clindamycin (CL:2 μg), and nitrofurantoin (F:30 μg) were used for Gram-positive bacteria while all but, erythromycin (E:15 μg), CL (2 μg), nitrofurantoin (F:30 μg), cephalothin (*CF*:30 μg), cefoxitin (FOX:30 μg), and CL (2 μg), were used for Gram-negative bacterial species. *E. coli* American type culture collection *25922 (ATCC 25922)* and *S. aureus* (*ATCC25923*) were used as quality control organisms for Gram-negative and Gram-positive organisms, respectively.

Isolates resistant to at least one in three or more antibiotic categories were considered multi-drug resistant (MDR) and being resistant to at least one to two or fewer antibiotic categories were considered extensive drug resistant (XDR), while those resistant to all antibiotic categories tested were considered pan-drug-resistant (PDR) (47, 48). The multiple antibiotic resistance index (MARI) was calculated as MARI = a/b, which is the ratio of the number of antibiotics to which an isolate is resistant (a) to the total number of antibiotics to which an isolate is tested (b) (49).

### Ethical consideration

The Institutional Research Ethics Review Board of the College of Health Sciences (IRERB Minutes Ref No.: AAUMF 03–008) and Aklilu Lemma Institute of Pathobiology (IRERB Minutes Ref No.: ALIPB IREC/86/14/22) Addis Ababa University reviewed the protocol and provided ethical clearance letter.

Comprehensive explanations were given to the study participants about the objective of the study in a private room prepared for interview, examination, and chart review before obtaining written consent. The study participants were assured not to participate and/or discontinue the interview at any point in time. The data collected was stored in a locker, which can only be accessed by the principal investigator. We assured and implemented that those found culture positive for UTIs were treated for the infection under the follow-up institution and assigned health care provider.

### Data management and analysis

The collected data were entered into Epi-data version 3.1 and exported to SPSS version 23 for analysis. Figures and tables were used to summarize the data. To measure the association of variables with the occurrence of UTIs, we used univariable and multivariable logistic regression analyses. Crude odds ratios (CORs) were obtained from the univariable regression analysis, while adjusted odds ratios (AORs) were derived from the multivariable regression analysis. The results from the final models were presented as AOR with a 95% CI, with association determined at a threshold of a *p*-value of <0.05.

## Results

### Socio-demographic characteristics

Among a total of 688 PLHIV tested for UTIs with a response rate of 98.1%, the majority (*n* = 349; 50.7%) fell within the age group 35–49 years, and *n* = 179 (26.0%) fell within the age group ≥50 years or older (Table 1). The median age was 42.0 years, with an interquartile range of 35.0 and 50.0 years old. The majority were females (*n* = 474; 68.9%), urban residents (*n* = 467; 67.9%), and unmarried (*n* = 365; 53.1%; Table 1).

**Table 1:** Background characteristics of study participants and association with UTIs, *N* = 688.

Variables	No. tested for UTIs: 688	+ve for UTIs 144(20.9%)	COR(95%CI)	AOR(95%CI)	*p*-value
Age					
18–34	160(23.3)	23(14.4)	0.57(0.32, 0.99)*	0.78(0.42, 1.45)	0.42
35–49	349(50.7)	80(22.9)	1.00(0.65, 1.54)	1.25(0.77, 2.03)	0.37
≥50	179(26.0)	41(22.9)	1		
Sex					
Male	214(31.1)	54(25.2)	1.44(0.98, 2.12)	1.38(0.87, 2.18)	0.18
Female	474(68.9)	90(19.0)	1		
Residence					
Urban	467(67.9)	104(22.3)	1.30(0.86, 1.95)	1.33(0.83, 2.12)	0.23
Rural	221(32.1)	40(18.1)	1		
Religion					
Christian	563(81.8)	121(21.5)	1.21(0.74, 1.99)	1.24(0.71, 2.18)	0.45
Muslim	125(18.2)	23(18.4)	1		
Marital Status					
Married	323(46.9)	74(22.9)	1.25(0.87, 1.81)	0.99(0.66, 1.50)	0.97
Unmarried	365(53.1)	70(19.2)	1		
Educational Status					
Illiterate	140(20.3)	28(20.0)	0.71(0.40, 1.26)	0.77(0.39, 1.53)	0.45
Secondary School	421(61.2)	83(19.7)	0.70(0.44, 1.11)	0.75(0.44, 1.29)	0.30
College and Above	127(18.5)	33(26.0)	1		
Occupational Status					
Unemployed	335(48.9)	69(20.6)	0.96(0.67, 1.39)	1.28(0.83, 1.98)	0.26
Employed	353(51.1)	75(21.3)	1		
Income per Month					
<3,000 EB	291(42.3)	55(18.9)	0.81(0.55, 1.18)	0.83(0.54, 1.28)	0.40
≥3,000 EB	397(57.7)	89(22.4)	1		
Frequency of Vaginal Douching /Perineal care per Day					
Not practicing	92(13.4)	26(28.3)	1.53(0.89, 2.62)	1.31(0.70, 2.46)	0.41
< Three times	313(45.5)	60(19.2)	0.92(0.62, 1.38)	0.85(0.54, 1.33)	0.47
≥ Three times	283(41.1)	58(20.5)	1		
History of Comorbidity with Chronic Diseases					
Comorbid	169(24.6)	48(28.4)	1.75(1.17, 2.61)*	1.52(0.71, 3.28)	0.28
Not comorbid	519(75.4)	96(18.6)	1		
Previous History for Chronic Diseases (169)					
Diabetic Mellitus	34(20.1)	12(35.3)	2.16(1.04, 4.47)*	1.50(0.58, 3.88)	0.41
Hypertension	36(21.3)	9(25.0)	1.28(0.59, 2.78)	0.91(0.34, 2.46)	0.86
UTIs	73(43.2)	21(28.8)	1.62(0.94, 2.78)	0.87(0.36, 2.10)	0.75
Renal Caliculi	26(15.4)	7(26.9)	1.41(0.58, 3.43)	0.46(0.15, 1.44)	0.18
Manifestation of UTIs Symptoms currently					
Yes	52(7.6)	36(69.20)	11(5.89, 20.53)*	11.57(5.83, 22.97)	<0.01*
No	636(92.4)	108(17.00)	1	1	

Unemployed includes house wife, student, farmer, daily laborers, and merchants; unmarried includes widowed, living together, divorced, and single; Christianity includes orthodox, protestant, and catholic; secondary education level includes primary level also, illiterate includes both able to write and read, and unable to write and read, comorbidity includes diabetes mellitus, hypertension, renal caliculi, and previous history of UTIs; 1(Reference), *(statistically significant at *p* > 0.05), urinary tract infections (UTIs), Ethiopian birr (EB).

A total of 313 participants (45.5%) reported that they were practicing vaginal/perineal care less than three times a day. Approximately one-fourth (*n* = 169; 24.6%) had comorbidities such as hypertension (*n* = 36; 5.2%), diabetes (*n* = 34; 4.9%), renal calculi (*n* = 26; 3.8%), or a history of previous UTIs (*n* = 73; 10.6%). During the course of this study, only 52 participants (7.6%) had symptoms associated with UTIs, such as urgency, frequent urination, and dysuria during micturition. Over one-third (*n* = 291; 42.3%) were earning a monthly income of less than 3,000 Ethiopian Birr (Table 1).

A total of 218 participants (31.7%) had a history of receiving anti-tuberculosis drugs, of which 84 (38.5%) were cured, while the remaining were still on treatment. Moreover, substantial (*n* = 51; 7.4%) of the study participants were receiving zidovudine-containing antiretroviral drug regimens (Table 2). In total, 59 (8.6%) of the study participants had a body mass index of less than 18.5wt/ht^2^, implying undernutrition (Table 2). Furthermore, the majority of the study participants 645(93.7%) were found under T1 World Health Organization clinical staging of HIV/AIDS, which implies that the viral load of these PLHIV was significantly reduced after starting the ART drugs (Table 2).

**Table 2:** Clinical characteristics and association with the occurrence of UTIs, *N* = 688.

Variables	No. tested for UTIs: 688	+ve for UTIs 144(20.9%)	COR(95%CI)	AOR(95%CI)	*p*-value
HIV RNA					
≥ 200 copies/ml	12(1.7)	9(75.0)	12.02(3.21, 45.01)*	12.24(3.24, 46.20)	<0.01*
<200 copies/ml	676(98.3)	135(20.0)	1		
WHO HIV Clinical Staging					
T1	645(93.7)	136(21.1)	0.27(0.02, 4.30)	0.20(0.01, 3.41)	0.27
III	41(6.0)	7(17.1)	0.21(0.01, 3.70)	0.17(0.01, 3.16)	0.23
IV	2(0.3)	1(50.0)	1		
ART Drug Regimen Receiving					
ZDV Containing	51(7.4)	12(23.5)	1.18(0.60, 2.31)	1.20(0.59, 2.43)	0.62
Non-ZDV Containing	637(92.6)	132(20.7)	1		
History of Anti-Tuberculosis Treatment					
Yes	218(31.7)	45(20.6)	0.98(0.66, 1.45)	1.07(0.40, 2.86)	0.89
No	470(68.3)	99(21.1)	1		
Outcome of the Anti-Tuberculosis Treatment					
Did not take	470(68.3)	100(21.3)	1.24(0.68, 2.27)	1.32(0.43, 4.03)	0.63
Improved	134(19.5)	29(21.6)	1.27(0.64, 2.54)	1.15(0.55, 2.37)	0.71
Cured	84(12.2)	15(17.9)	1		
Nutritional Status Based on BMI(wt/ht^2^)					
<18.5 wt/ht^2^	59(8.6)	17(28.8)	1.37(0.59, 3.16)	1.50(0.63, 3.55)	0.36
18.5–24.9 wt/ht^2^	406(59.0)	75(18.5)	0.77(0.39, 1.50)	0.80(0.40, 1.61)	0.54
25–29.9 wt/ht^2^	166(24.1)	39(23.5)	1.04(0.51, 2.13)	1.12(0.54, 2.35)	0.76
≥ 30wt/ht^2^	57(8.3)	13(22.8)	1	1	

UTIs, Urinary tract infections; T1, HIV stage after ART initiated, ZDV, zidovudine; WHO, World Health Organization; HIV, human immunodeficiency virus; wt/ht^2^, weight for height square; BMI, body mass index; wt/ht^2^, weight for height square; category of nutritional status: under nutrition (BMI: < 18.5wt/ht^2^), normal (BMI: 18.5–24.9wt/ht^2^), over nutrition (BMI: 25–29.9wt/ht^2^), and obese (BMI:≥ 30wh/ht^2^).

### Prevalence of UTIs and its associated factors

Out of a total of 688 study participants (*n* = 144; 20.9%) were found to have acquired UTIs confirmed by bacterial, culture. Of those who acquired UTIs, 36 participants (69.2%) were symptomatic, with the most prevalent symptom being dysuria (*n* = 14; 63.6%) (Table 1).

The majority of study participants with confirmed UTIs (*n* = 80; 22.9%), (*n* = 41; 22.9%) were aged 35–49, and at least 50 years, respectively. Similarly, among the total study participants with confirmed UTIs, 104 (22.3%) were urban residents, 90 (25.2%) were females, and 74 (22.9%) were married. In addition, a significant proportion of study participants with confirmed UTIs were not practicing perineal/vaginal douching (*n* = 26; 28.3%) and were comorbid with chronic diseases (*n* = 48; 28.4%; Table 1).

The presentation of clinical characteristics in relation to the prevalence of UTIs, among those who acquired UTIs (*n* = 144; 20.9%), a significant population (*n* = 9; 75.0%) had HIV RNA of ≥200 copies/ml, and the majority (*n* = 136; 21.1%) were in stage T1 of the World Health Organization clinical staging of HIV/AIDS. The higher (*n* = 12; 23.5%) proportions were from those who were receiving zidovudine-containing ART drug regimen of HIV treatment compared to those who were taking non-zidovudine-containing ART drug (Table 2).

Additionally, a sizable proportion (*n* = 29; 21.6%) of study participants who had UTIs were receiving anti-tuberculosis medication treatment. Similarly, an equivalent proportion (*n* = 100; 21.3%) of the study participants acquired UTIs and did not take anti-TB medications because they had not contracted tuberculosis. This suggests that individuals who took anti-TB medications regained competent immunity to the same extent as those who did not contract tuberculosis and did not take anti-TB medications (Table 2). Despite some factors, such as younger age (18–34 years old) [COR = 0.57(95% CI: 0.32, 0.99), *p* < 0.05], comorbidity with chronic diseases [COR = 1.75(95% CI: 1.17, 2.61), *p* < 0.02], diabetes mellitus [COR = 2.16(95% CI: 1.04, 4.47), *p* < 0.05], having the presence of symptomatic for UTIs [COR = 11(95% CI: 5.89, 20.53), *p* < 0.01], and having HIV RNA ≥ 200 copies/ml [COR = 12.02(95% CI: 3.21, 45.01), *p* < 0.01], were ruled out as factors affecting the occurrence of UTIs using univariable logistic regression analysis, results from multivariable logistic regression analysis revealed only HIV RNA of ≥200 copies/ml [AOR = 12.24 (95% CI, 3.24, 46.20), *p* < 0.01], and being symptomatic for UTIs during the study period [AOR = 11.57 (95% CI, 5.83, 22.97), *p* < 0.01] retained the association with the occurrence of UTIs (Tables 1, 2).

### Prevalence of bacterial isolates causing UTIs

**Figure 1:**
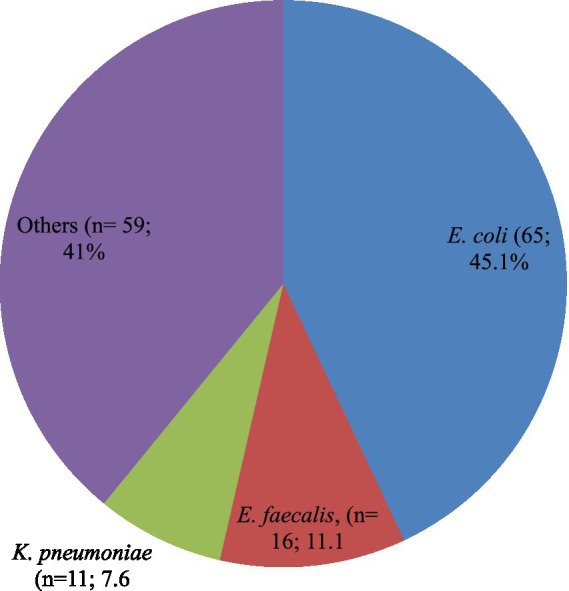
Proportion of bacterial species isolated among the PLHIV-acquired UTIs. Others includes: *Proteus mirabilis (n = 08; 5.6%)*; *Klebsiella oxytoca* (*n* = 06; 4.2%); *Enterobacter cloacae* (*n* = 04; 2.8%); *Enterobacter bugandensis* (*n* = 02; 1.4%); *Klebsiella aerogenes* (*n* = 01; 0.7%); *Fusobacterium nucleatum* (*n* = 01;0.7%); *Enterobacter amnigenus* (*n* = 01;0.7%); *Enterobacter asburiae* (*n* = 01;0.7%); *Cronobacter* sakazakii (*n* = 01;0.7%); *Alcaligenes faecalis* (*n* = 01;0.7%); *Salmonella* species (*n* = 01;0.7%); *Pseudomonas fulva* (*n* = 01;0.7%); *Staphylococcus aureus* (*n* = 08; 5.6%); *Bacillus cereus* (*n* = 06; 4.2%); *Enterococcus faecium* (*n* = 06; 4.2%); *Staphylococcus xylosus* (*n* = 04; 2.8%); *Staphylococcus sciuri* (*n* = 04; 2.8%); *Staphylococcus pasteuri* (*n* = 01; 0.7%); *Staphylococcus saprophyticus* (*n* = 01; 0.7%); and *Enterococcus hirae* (*n* = 01; 0.7%).

A total of 151 bacterial isolates were isolated from 144 study participants who had UTIs, which implies seven study participants who acquired UTIs were infected by more than one bacterial causal agent. The most frequently occurring bacterial isolates together were *E. coli* and *Bacillus cereus*. *E. coli* (*n* = 65; 45.1%) was the most common bacterial isolate identified followed by *E. faecalis* (*n* = 16; 11.1%), *K. pneumoniae* (*n* = 11; 7.6%). Likewise, a number of different rarely identified UTIs causing bacterial isolates accounting for a total of *n* = 59 (41%) includes *P. mirabilis* (*n* = 08; 5.6%), *Klebsiella oxytoca* (*n* = 06; 4.2%), *Enterobacter cloacae* (*n* = 04; 2.8%), *Enterobacter bugandensis* (*n* = 02; 1.4%), *E. faecium* (*n* = 06; 4.2%), *S. xylosus* (*n* = 04; 2.8%), and *S. sciuri* (*n* = 04; 2.8%). The remaining rarely causing UTIs accounts for *n* = 11 (7.7%) were *Klebsiella aerogenes, Fusobacterium nucleatum, Enterobacter amnigenus, Enterobacter asburiae, Cronobacter sakazakii, Alcaligenes faecalis, Salmonella species, Pseudomonas fulva, Staphylococcus pasteuri, S. saprophyticus, and E. hirae* (Figure 1).

Cross-tabulating the bacterial isolates rarely causes UTIs to selected study variables, the single bacterial isolates: *Salmonella species, Fusobacterium nucleatum, Klebsiella aerogenes, Pseudomonas fulva, Alcaligenes faecalis, Cronobacter sakazakii, Enterobacter asburiae, Enterobacter amnigenus, Staphylococcus pasteuri, and E. hirae*, were identified from study participants with older ages (35 years or older), living in urban areas, having history of chronic illness such as diabetes mellitus, hypertension, renal caliculi, and previous history of UTIs, no/less frequently practicing vaginal or perineal care, and currently exhibiting symptoms of UTIs. Conversely, *Alcaligenes feacalis, Fusobacterium nucleatum, Enterobacter asburiae, Enterobacter amnigenus*, and *E. hirae* were identified among asymptomatic study participants for UTIs.

Additionally, of the bacterial isolates that were found causing UTIs in this study, approximately one-third (*n* = 104; 68.9%) were Gram-negative, with the remainder being Gram-positive (Figure 1).

### Antibiotic resistance pattern and multiple antibiotic resistance index

The study revealed, that among Gram-negative bacterial isolates, all *K. pneumoniae, Enterobacter cloacae*, and *Enterobacter bugandensis* isolates were resistant to amoxicillin + clavulanic acid. All isolates of *Enterobacter bugandensis* were resistant to ciprofloxacin, azithromycin, and gentamicin. All *P. mirabilis* isolates were resistant to amoxicillin (Table 3; Supplementary Table 1).

**Table 3:** Antimicrobial susceptibility profile of bacterial strains isolated from urine culture-positive study participants, *N* = 151.

Bacterial isolates	% Resistant																	
Gram-negative	GM	AN	NA	C	CIP	CRO	TE	AMC	AZM	SXT	S	AMX	FOX	K	F	ERY	CL	CF
	%	%	%	%	%	%	%	%	%	%	%	%	%	%	%	%	%	%
*E. coli; N* = 65	67.7	70.8	64.6	26.2	61.5	13.8	50.8	23.1	69.2	29.2	52.3	36.9	NT	NT	NT	NT	NT	NT
*K. pneumoniae; N = 11*	18.2	9.1	36.4	27.3	45.5	54.5	45.5	100	18.2	63.6	45.5	100	NT	NT	NT	NT	NT	NT
Other; *N* = 28	57.1	28.6	28.6	35.7	35.7	32.1	25.0	35.7	25.0	32.1	46.4	82.1	NT	NT	NT	NT	NT	NT
Gram-positive																		
*E. faecalis; N = 16*	100	100	100	100	100	100	0.0	100	93.7	100	100	100	100	100	37.5	0.0	100	100
Other; *N* = 31	41.9	41.9	51.6	51.6	67.7	45.2	29.0	28	35.5	45.2	54.8	100	83.9	77.4	58.1	41.9	90.3	67.7

Resistant also includes a moderate level of resistance; Abbreviations used for antibacterial disks: GM, gentamicin; AN, amikacin; NA, nalidixic acid; C, chloramphenicol; CIP, ciprofloxacin; CRo, ceftriaxone; TE, tetracycline; AMC, amoxicillin + clavulanic acid; AZM, azithromycin; SXT, sulfamethoxazole + trimethoprim; S, streptomycin; AMX, amoxicillin; CL, clindamycin; F, nitrofurantoin; K, kanamycin; ERY, erythromycin; FOX, cefoxitin; CF, cephalothin; NT, not tested. Other Gram-negative bacteria include Proteus mirabilis, *N* = 08, Klebsiella oxytoca, *N* = 06, Enterobacter cloacae, *N* = 04, Enterobacter bugandensis, *N* = 02, Klebsiella aerogenes, *N* = 01, Fusobacterium nucleatum, *N* = 01, Enterobacter amnigenus, *N* = 01, Enterobacter asburiae, *N* = 01, Cronobacter sakazakii*, N = 01,* Alcaligenes faecalis, *N* = 01, Salmonella species, *N* = 01, and Pseudomonas fulva, *N* = 01, while other Gram-positive bacteria include: Staphylococcus aureus, *N* = 08, Bacillus cereus, *N* = 06, Enterococcus faecium, *N* = 06, Staphylococcus xylosus, *N* = 04, Staphylococcus sciuri, *N* = 04, Staphylococcus pasteuri, *N* = 01, Staphylococcus saprophyticus, *N* = 01, Enterococcus hirae, *N* = 01.

All eight rarely isolated Gram-negative bacteria associated with UTIs, namely, *Klebsiella aerogenes*, *Fusobacterium nucleatum*, *Enterobacter amnigenus*, *Enterobacter asburiae*, *Cronobacter sakazakii*, *Alcaligenes faecalis*, *Salmonella* species, and *Pseudomonas fulva*, were resistant to amoxicillin. Additionally, four of these rare isolates, namely, *Enterobacter amnigenus*, *Enterobacter asburiae*, *Alcaligenes faecalis*, and *Salmonella* species, demonstrated resistance to amoxicillin with clavulanic acid as well as other broad-spectrum antibiotics, including ciprofloxacin, ceftriaxone, azithromycin, gentamicin, and nalidixic acid (Table 3; Supplementary Table 1).

Over half of the *E. coli* isolates demonstrated resistance to multiple antibiotics: gentamicin (*n* = 44; 67.7%), amikacin (*n* = 46; 70.8%), nalidixic acid (*n* = 42; 64.6%), ciprofloxacin (*n* = 40; 61.5%), and azithromycin (*n* = 45; 69.2%). Additionally, a significant proportion of *K. pneumoniae* isolates were resistant to amoxicillin (*n* = 11; 100%), amoxicillin + clavulanic acid (*n* = 11; 100%), ceftriaxone (*n* = 6; 54.5%), and sulfamethoxazole + trimethoprim (*n* = 7; 63.6%; Table 3; Supplementary Table 1).

All isolates of *E. faecalis* (*n* = 16; 100%), *E. faecium* (*n* = 6; 100%), and the single *E. hirae* isolate exhibited resistance to several antibiotics, including gentamicin, amikacin, nalidixic acid, chloramphenicol, ciprofloxacin, amoxicillin, amoxicillin + clavulanic acid, sulfamethoxazole + trimethoprim, streptomycin, cefoxitin, kanamycin, clindamycin, and cephalothin. Additionally, all *E. faecalis* isolates (*n* = 16; 100%) were resistant to ceftriaxone, while only one *E. faecium* isolate (16.7%) showed resistance to ceftriaxone. None of the *E. hirae* isolates were resistant to ceftriaxone (Table 3; Supplementary Table 1).

All isolates of *S. xylosus* (*n* = 4; 100%), along with the single isolates of *S. pasteuri* and *S. saprophyticus*, were resistant to cefoxitin, amoxicillin, and amoxicillin + clavulanic acid. Additionally, these bacteria exhibited resistance to several broad-spectrum antibiotics, including amikacin, ciprofloxacin, ceftriaxone, erythromycin, kanamycin, clindamycin, and cephalothin (Table 3; Supplementary Table 1).

All *S. aureus* isolates were resistant to cefoxitin, amoxicillin, amoxicillin + clavulanic acid, and clindamycin. Additionally, over half of these isolates were resistant to ciprofloxacin, ceftriaxone, kanamycin, and cephalothin. These resistance patterns indicate that all *S. aureus* isolates in the study were methicillin-resistant, highlighting a significant challenge in treating infections caused by these bacteria (Table 3; Supplementary Table 1).

All bacterial isolates in the study were resistant to at least one antibacterial agent, with the highest multiple antimicrobial resistance index (MARI) observed among *E. faecalis*, *S. xylosus*, and *E. coli*. This indicates significant variability in ABR among the isolates (Supplementary Table 1).

A total of 138 isolates (91.4%) were resistant to at least one antibiotic in three or more categories, indicating MDR. Of these, 43 isolates (31.2%) were resistant to all but two or three of the antibiotic categories tested, classifying them as XDR. The high variability in the MARI among the isolates that developed MDR and XDR highlights a concerning trend in the increasing complexity and capriciousness of ABR (Supplementary Table 1).

## Discussion

Of the 688 study participants, 144 (20.9%) had culture-confirmed UTIs, with most being asymptomatic. This finding aligns with study from Ethiopia (18%) (22), Nigeria (19.3%) (50), and Tanzania (21.4%) (51). However, it is higher than other findings from Ethiopia, which range from 10.3 to 14.1% (4, 26, 28). Conversely, our study’s UTI prevalence is lower than the findings from South Africa (48.7%) (9), and another study from Nigeria (57.3%) (19). The observed differences could be attributed to several factors, including geographic location, sample size, immune status, and the level of adherence to ART drugs among the study participants.

Higher HIV RNA copies and the manifestation of UTI symptoms were found to be positively associated with the occurrence of UTIs. The most frequently identified bacterial uropathogen was *E. coli*, followed by *E. faecalis* and *K. pneumoniae*. Additionally, several rare UTI-causing bacterial isolates were identified, which exhibited high levels of ABR.

The link between higher levels of HIV RNA copies and the occurrence of UTIs highlights the intricate relationship between viral load and susceptibility to opportunistic infections, including UTIs. Elevated viral load often correlates with higher viral replication and a more active HIV infection, leading to the progressive depletion of CD4 cells. This results in a weakened immune system, increasing the risk of opportunistic infections. Various factors can influence this susceptibility, such as co-infections with other viruses, genetic factors, geographic location, lifestyle choices, nutrition, stress, and pregnancy, all of which contribute to a greater risk of acquiring infections such as UTIs (2).

Furthermore, the link between clinical symptoms of UTIs and the occurrence of UTIs may be attributed to the irritation of the bladder trigone or urethra. This irritation causes the bladder to contract, resulting in more frequent and painful urination (52).

The distribution of bacterial isolates causing UTIs revealed in the current study shows approximately three-fourths (n = 104; 68.9%) were Gram-negative organisms. This distribution aligns with study findings from Ethiopia (28), South Africa (9), and Uganda (53). However, studies from other regions in Nigeria (19) and India (54) reported Gram-positive bacteria as the most prevalent UTI-causing bacteria among PLHIV. This variation could be due to differences in geographic location, characteristics of the study population, and the laboratory methodologies used for bacterial isolation in the different regions.

The frequent isolation of *E. coli* could be due to certain strains, known as uropathogenic *E. coli* (UPEC), possessing specialized virulence factors. These include adhesins (*fim* and *pap operons*), *toxins* (*hemolysin* and *cytotoxic-necrotizing* factors), and *siderophores* (*fyuA gene*), which facilitate their ability to cause UTIs. These virulence factors enable UPEC to adhere to the urinary tract epithelium, evade the host immune response, and acquire essential nutrients, thereby increasing their pathogenic potential (2). This finding aligns with studies conducted globally (55), as well as in Ethiopia (28), Nigeria (56), Uganda (53), and Croatia (57). However, contrary to our study, findings from Nigeria (19), Ghana (24), and India (54) revealed that the most frequently isolated bacterial species causing UTIs was *S. aureus*.

The identification of *E. faecalis* as the second most frequent bacterial species in this study, with a higher prevalence of ABR, could be due to its significant impact on vulnerable populations, such as those living with HIV (58). This pathogen’s resilience and adaptability make it particularly problematic in immunocompromised individuals, contributing to its prominence in such settings (58). This finding is consistent with studies from Tanzania (27) and Croatia (57), raising concerns regarding public health implications, especially in areas with limited access to effective antibiotics. The prevalence of *E. faecalis* and its resistance to antibiotics highlights the need for improved infection control measures and the development of more effective treatment strategies in such regions.

The less frequently isolated bacterial species causing UTIs reported in the current study raise concerns about the emergence of new pathogens contributing to UTI occurrence. This issue is significant for global public health, as it highlights the need for ongoing vigilance and research. The scientific community needs to investigate these emerging bacteria, understand their pathogenic mechanisms, and develop effective treatment and preventive strategies to address the evolving landscape of UTI pathogens.

The higher prevalence of bacterial uropathogens, including rarely identified pathogens causing UTIs, as revealed by studies in the region, highlights growing concerns about increasing ABR globally. This escalating threat of antimicrobial resistance (AMR) poses a substantial challenge to public health, particularly among PLHIV in sub-Saharan African countries where resources are limited (59). Addressing this issue requires a multi-faceted approach: enhanced surveillance, antibiotic stewardship, infection control measures, research and development, public awareness and education, strengthening healthcare systems, and global collaboration.

The finding that all bacterial isolates were resistant to at least one antibacterial agent, along with the high levels of MDR, underscores the gravity of the problem. This situation is concerning as it implies limited treatment options, an increased risk of complications, and significant public health implications.

The higher prevalence of ABR observed in the current study among *E. coli* isolates to various antibiotics: ciprofloxacin (61.5%), azithromycin (69.2%), and nalidixic acid (64.6%). This finding aligns with studies from Nigeria (50) and Ethiopia (4), indicating a broadening issue of AMR.

However, the variation in resistance patterns, such as the lower resistance rate reported in Harar, Ethiopia (20.5%) (22), highlights the multifactorial nature of AMR. Differences in resistance patterns across regions can be attributed to factors such as local antibiotic prescribing practices, infection control measures, healthcare infrastructure, and the prevalence of resistance genes. This highlights the need for region-specific strategies to monitor and address resistance trends effectively.

The observed high prevalence of ABR among *P. mirabilis* to various antibiotics, such as amoxicillin, amoxicillin+clavulanic acid, and gentamicin, aligns with study findings from Nigeria (50) and Ethiopia (22). This widespread resistance poses significant challenges for treatment. However, the contrasting results from India (60) highlight the variability of antibiotic resistance patterns across different regions. This variability highlights the importance of local surveillance and tailored treatment strategies to effectively manage and address antibiotic resistance in different settings.

The World Health Organization has identified MRSA as a significant pathogen that poses serious health risks (61). MRSA alone was responsible for over 100,000 deaths in 2019 (62). The current study found that all *S. aureus* isolates were resistant to cefoxitin, indicating a 100% prevalence of MRSA. This rate is notably higher than the 27.9% prevalence reported in Ireland (63).

The high prevalence of MRSA in this study may be attributed to factors such as the inappropriate use of antibiotics, which can drive the development and spread of resistant strains. This highlights the urgent need for improved antibiotic stewardship and infection control measures.

This study highlights a crucial aspect that could further enhance its comprehensiveness, investigating other potential causative agents of UTIs beyond bacterial isolates. By including viruses and fungi, researchers could gain a more complete understanding of the prevalence, etiology, and management of UTIs among PLHIV. Such an approach would provide a broader perspective on the range of pathogens involved, potentially leading to more effective diagnostic and treatment strategies.

## Conclusion

The current study revealed a high prevalence of UTIs and elevated levels of ABR as compared to similar studies in other regions. Independent predictors for UTI occurrence identified in the study include higher HIV RNA copies and the manifestation of UTI symptoms during the study period. The predominant bacterial isolates in this population were *E. coli*, *K. pneumoniae*, and *E. faecalis*. This finding highlights the need for targeted surveillance, raising public awareness, integrating UTI screening programs for PLWH, establishing measures for responsible antibiotic use and antibiotic stewardship programs, and tailoring treatment strategies to address the unique challenges posed by these pathogens and resistance patterns in the study population. The study also found several rare UTI-causing bacterial species which need to be investigated.

## Data Availability

The data presented in the study are deposited in the Mendeley data repository. This data can be found here: https://data.mendeley.com/datasets/cg8y6cvs3f/1, DOI: 10.17632/cg8y6cvs3f.1.

## Glossary

A/A: Acid/acid
A/K: Acid/base
A/R: Acid/red
AIDS: Acquired immunodeficiency syndrome
ART: Antiretroviral therapy
ATCC: American Type Culture Collection
CLED: Cystine–lactose–electrolyte-deficient agar
EMB: Eosin-methylene blue
HIV: Human immunodeficiency virus
LIA: Lysine iron agar
MARI: Multiple antibiotic resistance index
MDR: Multidrug resistance
*MRSA*: Methicillin-resistant *Staphylococcus aureus*
PDR: Pan-drug resistance
PLHIV: People living with HIV
RNA: Ribonucleic acid
SPP: Species
TSA: Triple sugar agar
UPEC: Uropathogenic *E. coli*
UTIs: Urinary tract infections
WHO: World Health Organization
XDR: Extensive drug resistance
