# Supplementary material for: Urinary tract infection among people living with human immunodeficiency virus attending selected hospitals in Addis Ababa and Adama, central Ethiopia
Source: Front Public Health. 2024 Sep 4;12:1394842. doi: 10.3389/fpubh.2024.1394842 (PMC11408745; doi:10.3389/fpubh.2024.1394842)
Supplement: Supplementary file 1 [file Table_1.docx]

| **Supplementary Table 1: Antibiotic Resistance Pattern of Bacterial Uro-Pathogens, N = 151** | | | | | | | | | | |
| --- | --- | --- | --- | --- | --- | --- | --- | --- | --- | --- |
| **Gram-negative Bacteria** | | | | | | | | | | |
|  | | Resistance Pattern | NoRP | | | TNR | | | MARI | |
| *E. coli*  N = 65 | | **GM,AN,NA,CIP,TE,AZM,S* | | 03 | | | 07 | | 0.58 | |
|  |  | **GM,AN,NA,AMX,TE,S* | | 03 | | | 06 | | 0.50 | |
|  |  | **GM,AN,NA,CIP,AZM,S* | | 02 | | | 06 | | 0.50 | |
|  |  | **GM,NA,CIP,TE,AZM,S* | | 02 | | | 06 | | 0.50 | |
|  |  | **GM,AN,CIP,AZM,S* | | 02 | | | 05 | | 0.42 | |
|  |  | **GM,AN,NA,CIP,TE* | | 02 | | | 05 | | 0.42 | |
|  |  | **AN,NA,C,CIP,AZM,S* | | 01 | | | 06 | | 0.50 | |
|  |  | **AN,NA,C,AMX,AZM,AMC* | | 01 | | | 06 | | 0.50 | |
|  |  | **AN,NA,C,TE,AZM,S* | | 01 | | | 06 | | 0.50 | |
|  |  | **GM,C,AN,NA,AZM,S* | | 01 | | | 06 | | 0.50 | |
|  |  | **GM,NA,C,CIP,AZM,S* | | 01 | | | 06 | | 0.50 | |
|  |  | **GM,AN,TE,AZM,AMC,AMX* | | 01 | | | 06 | | 0.50 | |
|  |  | **GM,NA,CIP,TE,AZM* | | 01 | | | 05 | | 0.42 | |
|  |  | **GM,AN,NA,CIP,S* | | 01 | | | 05 | | 0.42 | |
|  |  | **GM,AN,NA,TE,AZM* | | 01 | | | 05 | | 0.42 | |
|  |  | **GM,AN,NA,TE,S* | | 01 | | | 05 | | 0.42 | |
|  |  | **GM,AN,NA,AZM,SXT* | | 01 | | | 05 | | 0.42 | |
|  |  | **GM,AN,CIP,AZM,SXT* | | 01 | | | 05 | | 0.42 | |
|  |  | **AN,NA,CIP,TE,AZM* | | 01 | | | 05 | | 0.42 | |
|  |  | **AN,NA,C,CIP,TE* | | 01 | | | 05 | | 0.42 | |
|  |  | **AMX,CIP,CRO,AZM,AMC* | | 01 | | | 05 | | 0.42 | |
|  |  | **GM,C,AMX,S,CIP* | | 01 | | | 05 | | 0.42 | |
|  |  | **GM,NA,AZM,S* | | 01 | | | 04 | | 0.33 | |
|  |  | **GM,AN,NA,C* | | 01 | | | 04 | | 0.33 | |
|  |  | **GM,NA,TE,S* | | 01 | | | 04 | | 0.33 | |
|  |  | **GM,AN,NA,SXT* | | 01 | | | 04 | | 0.33 | |
|  |  | **NA,CIP,AZM,SXT* | | 01 | | | 04 | | 0.33 | |
|  |  | **GM,C,AZM,S* | | 01 | | | 04 | | 0.33 | |
|  |  | **AN,CIP,TE,AZM* | | 01 | | | 04 | | 0.33 | |
|  |  | **GM,C,AMX,AMC* | | 01 | | | 04 | | 0.33 | |
|  |  | **GM,TE,AZM* | | 01 | | | 03 | | 0.25 | |
|  |  | **GM,NA,AZM* | | 01 | | | 03 | | 0.25 | |
|  |  | **AN,C,AMX* | | 01 | | | 03 | | 0.25 | |
|  |  | **C,CIP,TE* | | 01 | | | 03 | | 0.25 | |
|  |  | *GM,SXT,S* | | 01 | | | 03 | | 0.25 | |
|  |  | *GM,AN,AMX* | | 01 | | | 03 | | 0.25 | |
|  |  | *GM,AN,AZM* | | 01 | | | 03 | | 0.25 | |
|  |  | *GM,CIP,S* | | 01 | | | 03 | | 0.25 | |
|  |  | *GM,CIP* | | 01 | | | 02 | | 0.17 | |
|  |  | *AN* | | 01 | | | 01 | | 0.08 | |
|  |  | **GM,AN,NA,AMX,CIP,CRO,AZM,SXT,S,AMC*** | | 01 | | | 10 | | 0.83 | |
|  |  | **GM,AN,NA,C,CIP,CRO,TE,AZM,SXT,S*** | | 01 | | | 10 | | 0.83 | |
|  |  | **GM,NA,C,AMX,CIP,TE,AZM,SXT,S,AMC*** | | 01 | | | 10 | | 0.83 | |
|  |  | **GM,AN,NA,AMX,CIP,AZM,SXT,S,AMC*** | | 01 | | | 09 | | 0.75 | |
|  |  | **GM,AN,AMX,CIP,TE,AZM,SXT,S,AMC*** | | 01 | | | 09 | | 0.75 | |
|  |  | **GM,AN,AMX,CIP,CRO,AZM,SXT,S,AMC*** | | 01 | | | 09 | | 0.75 | |
|  |  | **AN,NA,CRO,TE,AZM,SXT,S,AMX,AMC*** | | 01 | | | 09 | | 0.75 | |
|  |  | **AN,NA,AMX,CIP,TE,AZM,SXT,AMC*** | | 01 | | | 08 | | 0.67 | |
|  |  | **AN,NA,AMX,CIP,TE,AZM,S,AMC* | | 01 | | | 08 | | 0.67 | |
|  |  | **AN,NA,CIP,CRO,TE,AZM,SXT,S*** | | 01 | | | 08 | | 0.67 | |
|  |  | **AN,NA,AMX,CIP,SXT,S,AMC,AZM* | | 01 | | | 08 | | 0.67 | |
|  |  | **AN,NA,C,AMX,CIP,CRO,S,AZM*** | | 01 | | | 08 | | 0.67 | |
|  |  | **GM,AN,NA,CIP,CRO,TE,AZM,SXT*** | | 01 | | | 08 | | 0.67 | |
|  |  | **AN,NA,AMX,CIP,TE,AZM,AMC,SXT*** | | 01 | | | 08 | | 0.67 | |
|  |  | **AN,NA,C,AMX,TE,AZM,AMC* | | 01 | | | 07 | | 0.58 | |
|  |  | **AN,AMX,CRO, CIP,TE,AZM,SXT*** | | 01 | | | 07 | | 0.58 | |
|  |  | **C,CIP,CRO,TE,AZM,SXT,AMX*** | | 01 | | | 07 | | 0.58 | |
| *Klebsiella pneumoniae;*  N = 11 | | **GM,AN,TE,AMC,AZM,SXT,S,AMX* | | 01 | | | 08 | | 0.67 | |
|  |  | **NA,C,CIP,CRO,TE,AMC,AMX* | | 01 | | | 07 | | 0.58 | |
|  |  | **NA,CIP,CRO,AMC,SXT,S,AMX* | | 01 | | | 07 | | 0.58 | |
|  |  | **NA,CIP,TE,AMC,SXT,S,AMX* | | 01 | | | 07 | | 0.58 | |
|  |  | **CRO,TE,AZM,AMC,SXT,S,AMX* | | 01 | | | 07 | | 0.58 | |
|  |  | **GM,NA,CIP,CRO,AMC,AMX* | | 01 | | | 06 | | 0.50 | |
|  |  | **C,CRO,TE,SXT,AMC,AMX* | | 01 | | | 06 | | 0.50 | |
|  |  | **C,SXT,S,AMC,AMX* | | 01 | | | 05 | | 0.42 | |
|  |  | *CIP,AMC,AMX* | | 01 | | | 03 | | 0.25 | |
|  |  | *SXT,AMC,AMX* | | 01 | | | 03 | | 0.25 | |
|  |  | *CRO,AMC,AMX* | | 01 | | | 03 | | 0.25 | |
| *Proteus mirabilis*;  N = 08 | | **GM,CRO,AMC,AMX,S* | | 02 | | | 05 | | 0.42 | |
|  |  | **AN,CRO,AMC,AMX,S* | | 01 | | | 05 | | 0.42 | |
|  |  | **GM,CRO,AMC,AMX* | | 01 | | | 04 | | 0.33 | |
|  |  | *CRO,AMXC,AMX* | | 01 | | | 03 | | 0.25 | |
|  |  | *GM,AMC,AMX* | | 01 | | | 03 | | 0.25 | |
|  |  | **GM,AN,CRO,AMC,AMX,S* | | 01 | | | 06 | | 0.50 | |
|  |  | **GM,AN,C,TE,AZM,SXT,AMX* | | 01 | | | 07 | | 0.58 | |
| *Klebsiella oxytoca;*  N = 06 | | **NA,C,AMC,AMX* | | 02 | | | 04 | | 0.33 | |
|  |  | *NA,C* | | 01 | | | 02 | | 0.17 | |
|  |  | **GM,CIP,TE,AZM,SXT,AMX* | | 01 | | | 06 | | 0.50 | |
|  |  | **NA,C,CIP,SXT,S,AMX* | | 01 | | | 06 | | 0.50 | |
|  |  | **AN,NA,C,AMC,S,AMX* | | 01 | | | 06 | | 0.50 | |
| *Enterobacter cloacae;*  *N = 04* | | **GM,C,CIP,CRO,AMC,SXT,AMX,S* | | 01 | | | 08 | | 0.67 | |
|  |  | **TE,AMC,SXT,AMX,S* | | 01 | | | 05 | | 0.42 | |
|  |  | **TE,AMC,SXT,AMX* | | 01 | | | 04 | | 0.33 | |
|  |  | **NA,C,AMC,AMX* | | 01 | | | 04 | | 0.33 | |
| *Enterobacter bugandensis;*  *N = 02* | | **GM,AMC,AN,CIP,AMX,AZM* | | 01 | | | 06 | | 0.50 | |
|  |  | **GM,AMC,CIP,AMX,AZM* | | 01 | | | 05 | | 0.42 | |
| *Klebsiella aerogenes; N = 01* | | **CIP,TE,AMC,SXT,S,AMX* | | 01 | | | 06 | | 0.50 | |
| *Fusobacterium nucleatum;*  *N = 01* | | **GM,CIP,SXT,AMX* | | 01 | | | 04 | | 0.33 | |
| *Enterobacter amnigenus;*  *N = 01* | | **GM,AMC,AMX,CIP,AZM,* | | 01 | | | 05 | | 0.42 | |
| *Enterobacter asburiae; N = 01* | | **GM,TE,AN,NA,S,AZM AMC,AMX* | | 01 | | | 08 | | 0.67 | |
| Cronobacter sakazakii; N = 01 | | **GM,AN,NA,S,AZM,AMX* | | 01 | | | 06 | | 0.50 | |
| *Alcaligenes faecalis;* N = 01 | | **GM,TE,AMC,AN,CIP,NA,S,AMX* | | 01 | | | 08 | | 0.67 | |
| Salmonella species; N = 01 | | **AMC,CRO,C,AMX,S,AZM* | | 01 | | | 06 | | 0.50 | |
| *Pseudomonas fulva; N = 01* | | **C,CIP,TE,AMX* | | 01 | | | 04 | | 0.33 | |
| **Gram-positive Bacteria** | | | | | | | | | | |
| *Enterococcus faecalis;* N = 16 | **GM,AMC,CRO,C,AN,CIP,NA,SXT,AMX,S,AZM,FOX,K,CF,CL*** | | | | 09 | | | 15 | | 0.83 |
|  | **GM,AMC,CRO,C,AN,CIP,NA,SXT,AMX,S,AZM,FOX,K,CF,F,CL*** | | | | 06 | | | 16 | | 0.89 |
|  | **GM,AMC,CRO,C,AN,CIP,NA,SXT,AMX,S,FOX,K,CF,CL*** | | | | 01 | | | 14 | | 0.78 |
| *Staphylococcus aureus; N = 08* | **AN,NA,C,CIP,CRO,TE,AMX,S,FOX,K,CF,F,CL,AMC*** | | | | 01 | | | 14 | | 0.78 |
|  | **NA,C,CIP,CRO,TE,AMX,S,FOX,K,ERY,CL,AMC*** | | | | 01 | | | 12 | | 0.67 |
|  | **CIP,CRO,TE,AMX,FOX,K,CF,F,CL,AMC* | | | | 01 | | | 10 | | 0.56 |
|  | **NA,CIP,CRO,AMX,FOX,K,CF,F,CL,AMC* | | | | 01 | | | 10 | | 0.56 |
|  | **CRO,AMX,S,FOX,K,CF,ERY,CL,AMC* | | | | 01 | | | 09 | | 0.50 |
|  | **C,AMX,SXT,S,FOX,K,CF,ERY,CL,AMC* | | | | 01 | | | 09 | | 0.50 |
|  | **NA,CIP,CRO,AMX,FOX,K,CF,CL,AMC* | | | | 01 | | | 09 | | 0.50 |
|  | **GM,C,AMX,SXT,S,FOX,CF,ERY,CL,AMC* | | | | 01 | | | 09 | | 0.50 |
| *Bacillus cereus; N= 06* | **AN,NA,CIP,CRO,TE,AMX,SXT,FOX,K,CF,F,ERY,CL*** | | | | 01 | | | 12 | | 0.67 |
|  | **C,TE,AMC,S,AMX,SXT,FOX,K,F,ERY,CL* | | | | 01 | | | 10 | | 0.56 |
|  | **AMC,AZM,SXT,S,AMX,FOX,K,CF,F,CL* | | | | 01 | | | 10 | | 0.56 |
|  | **CIP,CRO,AMC,AZM,S,AMX,F,ERY,CL* | | | | 01 | | | 09 | | 0.50 |
|  | **GM,C,TE,AMC,AMX,AZM,S,FOX,CL* | | | | 01 | | | 08 | | 0.44 |
|  | **AMC,S,AMX,FOX,F,CL* | | | | 01 | | | 06 | | 0.33 |
| *Enterococcus faecium;* N=06 | **GM,C,AN,CIP,NA,SXT,S,AZM,AMC,AMX,FOX,K,CF,CL*** | | | | 03 | | | 12 | | 0.67 |
|  | **GM,C,AN,CIP,NA,SXT,S, AMC,AMX,F,FOX,K,CF,CL*** | | | | 01 | | | 12 | | 0.67 |
|  | **GM,C,AN,CIP,NA,SXT,S,AZM, AMC,AMX,FOX,K,CF,CL*** | | | | 01 | | | 11 | | 0.61 |
|  | **GM,CRO,C,AN,CIP,NA,SXT,S,AZM,FOX,K,CF,CL*** | | | | 01 | | | 13 | | 0.72 |
| *Staphylococcus xylosus;* N = 04 | **AN,NA,CIP,AMC,AMX,FOX,CF,K,CL* | | | | 01 | | | 09 | | 0.50 |
|  | **GM,AN,NA,CIP,CRO,TE,AMX,AMC,FOX,CF,K,ERY,CL*** | | | | 01 | | | 13 | | 0.72 |
|  | **GM,AN,NA,C,CIP,CRO,AZM,SXT,AMX,AMC,FOX,CF,F,K,ERY,CL*** | | | | 01 | | | 16 | | 0.89 |
|  | **GM,AN,C,CIP,TE,AZM,SXT,S,AMX,AMC,FOX,CF,F,K,ERY,CL*** | | | | 01 | | | 16 | | 0.89 |
| *Staphylococcus sciuri;* N = 04 | *AMC,AMX* | | | | 01 | | | 02 | | 0.11 |
|  | **AMX,K,F* | | | | 01 | | | 03 | | 0.17 |
|  | **NA,C,CIP,CRO,AMX,F,ERY,CL* | | | | 01 | | | 07 | | 0.39 |
|  | **GM,CIP,CRO,AMC,AMX,K,CF,ERY,CL* | | | | 01 | | | 09 | | 0.50 |
| *Staphylococcus Pasteuri; N = 01* | **TE,AMC,AMX,FOX,K,CL,CF* | | | | 01 | | | 07 | | 0.39 |
| *Staphylococcus saprophyticus;*  *N = 01* | **CIP,CRO,AMC,AMX,FOX,K,ERY,CF* | | | | 01 | | | 08 | | 0.44 |
| *Enterococcus hirae; N = 01* | **GM,AN,NA,C,CIP,AZM,AMC,AMX,SXT,S,FOX,K,F,CL,CF*** | | | | 01 | | | 15 | | 0.83 |
| Abbreviations; BS (Bacterial Isolates); (NoRP) Number of Resistance Pattern; TNR (Total Number of Antibiotics Resistant); MARI (Multiple Antibiotic Resistance Indexes), *(MDR: Multiple Drug Resistance), ** (XDR: Extensive Drug Resistance), GM(gentamicin); AN(Amikacin); NA(Nalidixic acid); C(Chloramphenicol); CIP(Ciprofloxacin); CRO(Ceftriaxone); TE(Tetracycline); AMC(Amoxicillin + clavulanic acid); AZM(Azithromycin); SXT(Sulphamethoxazole + Trimethoprim); S(Streptomycin), AMX(Amoxicillin); CL (Clindamycin); F(Nitrofurantoin), K(Kanamycin); ERY(Erythromycin); FOX(Cefoxitin); CF(Cephalothin). | | | | | | | | | | |
